# Drivers, scales and contexts of disaster risk: An editorial introduction

**DOI:** 10.4102/jamba.v8i2.270

**Published:** 2016-01-13

**Authors:** Lori Peek

**Affiliations:** 1 Department of Sociology, Colorado State University, United States

From 06–08 October 2014, I had the opportunity to attend the second biennial conference of the Southern Africa Society for Disaster Reduction in Windhoek, Namibia. The theme of that meeting was drivers, scales and contexts of disaster risk in the Southern Africa Development Community. The three-day event included morning plenary sessions, afternoon breakout paper sessions and evening social hours and networking events.

Those conference days were filled with lively debate and discussion. This dynamism of interchange was due, in part, to the diversity of the participants themselves: They hailed from all over the southern Africa region as well as from other parts of Africa, Europe, North America, Asia and beyond. Speakers included elected and appointed government officials, practitioners, graduate students, faculty members and many others. The sessions and associated events opened up the opportunity for long-standing collaborators as well as new colleagues and acquaintances to connect and to converse about common research interests and practical applications.

At the centre of that conference – as well as at the heart of this special issue of *Jàmbá: Journal of Disaster Risk Studies* – was a deep and abiding desire to understand the forces that generate disaster risk and the associated possibilities for reducing that risk across multiple scales and contexts.

In the pages of this issue, readers will find articles that use a variety of methodological approaches to explore disaster risk, disaster impact and risk reduction from numerous disciplinary and theoretical perspectives. Some of the papers offer in-depth and up-close qualitative investigations. Others include numerical portraits of various trends and patterns, drawn from surveys or secondary-data analysis. A select few draw on mixed-methods approaches to shed light on the topic of interest. Taken together, the papers explore the entire life cycle of disaster, from preparedness and mitigation activities to emergency response and short and longer-term recovery. The papers that assess the impact of disaster range from the individual to the institutional level.

The articles in this issue also vary in terms of the geographic scope of interest with some focusing on more rural and less heavily populated areas in the southern Africa region and others examining disaster risk-reduction activities in more suburban or urban localities. As a consequence, some of the contributors focus on rural farmers whilst others help us to understand the experience of city-dwelling populations. You will read about children and the elderly in these pages as well as about diverse family structures and cultures.

Whilst each individual paper makes its own distinct contribution to the empirical literature, there are also several important overarching themes worthy of noting here.

Firstly, the papers included herein help stretch the bounds of the term disaster. Papers explore the short and longer-term effects of both acute-onset disasters like floods and slower-onset, creeping disasters like droughts. They take on public-health emergencies associated with crises such as cholera and HIV-AIDS, respectively. Accidental or human-caused disasters generated as an unintended consequence of modern technological advances are also considered.

Secondly, all of the papers discuss the complex and heavily interconnected drivers of disaster. Environmental degradation, population growth and migration, unsustainable development, social and economic inequality, lack of political representation, cultural loss, changes in family structure and many other explanations are offered in the following pages. The authors are adept at placing disaster in historical context whilst also tracing change across time and space to help the reader understand how and when human actions turned hazards into disasters.

Thirdly, whilst most of the papers in this issue focus on one central disaster, I was struck by the vivid descriptions of associated cascading crises or other disasters that followed the initial event. The chain reactions of disaster that are described in these papers are important and worthy of further consideration. The authors clearly illustrate that disasters are rarely bound in time or in a singular geographic context. Instead, like waves in the ocean, the ripple effects of these events can and often do move across generations, across organisational and social contexts and throughout space. The diffuse and enduring nature of disaster, and the damage that these events can do in terms of achieving development goals, are points that the authors make in both explicit and implicit ways throughout the subsequent pages.

Fourthly, the papers strike an important balance in terms of including realistic (and sometimes devastating) representations of the toll that disasters can take on people and places while also describing household and community-based initiatives that have served to move the needle forward in terms of reducing disaster risk. Each paper ends with a recommendation or implications section, which helps the reader to understand not just what happened but what can be done in future to avoid calamity. These articles, therefore, should be of great interest, not just to other academics but to practitioners and policy makers as well.

Last October, I had the chance to hear many of these papers presented for the first time at the biennial conference of the Southern Africa Society for Disaster Reduction. Now, thanks to the dedicated work of the authors and the *Jàmbá* editorial staff, the readers of this journal can also share in the knowledge generated at the conference. For that, and for these excellent contributions, I am grateful.

